# Paired blood and brain tissue methylation biomarkers in focal cortical dysplasia

**DOI:** 10.1093/braincomms/fcaf277

**Published:** 2025-08-22

**Authors:** Ishant Khurana, Jean Khoury, Robyn M Busch, Ingmar Blümcke, Imad Najm, Assam El-Osta

**Affiliations:** Baker Heart and Diabetes Institute, Epigenetics in Human Health and Disease Program, Melbourne, Victoria 3004, Australia; Epilepsy Center, Neurological Institute, Cleveland Clinic, Cleveland, OH 44195, USA; Epilepsy Center, Neurological Institute, Cleveland Clinic, Cleveland, OH 44195, USA; Institute of Neuropathology, University Hospitals Erlangen, Erlangen 91054, Germany; Epilepsy Center, Neurological Institute, Cleveland Clinic, Cleveland, OH 44195, USA; Baker Heart and Diabetes Institute, Epigenetics in Human Health and Disease Program, Melbourne, Victoria 3004, Australia

**Keywords:** epilepsy, malformation of cortical development, focal cortical dysplasia, genetics, DNA methylation

## Abstract

Focal Cortical Dysplasia (FCD) is a common cause of drug-resistant epilepsy. These abnormalities arise during embryonic development and are challenging to classify due to their complex nature. The most recent classification update of FCD incorporates genetic and epigenetic results with other clinical data for the management of epilepsy associated with these lesions. Mutations in the mechanistic target of rapamycin pathway have been described in subtypes IIa and IIb of FCD. In this study, we aimed to study brain DNA methylation in human FCD samples and determine whether blood DNA methylation reflects epigenetic changes observed in brain tissue. We studied genome-wide methylation in 21 brain tissue samples (FCD; *n =* 13 and other pathologies; *n =* 8) resected from patients with medically intractable epilepsy along with matched blood samples from the same patients. These results were validated with 32 brain-blood matched samples. This study identified both unique and shared methylation signatures in brain and blood for FCD subty pes IIa and IIb and validated three methylation biomarkers (*Interleukin-1 receptor accessory protein*, *Homeodomain-interacting protein kinase 2*, and *Chronomodulin*) that differentiate these subtypes. Methyl-Binding Domain capture sequencing identified 676 551 methylated regions, covering 70% of cytosine–guanine dinucleotide sites in the genome. Adjustments for factors like age, gender, and disease duration were made before analysis. A total of 13 methylation biomarkers were identified for improved classification of FCD IIb from IIa. The three biomarkers showed high specificity and sensitivity, with an area under the curve score of 0.98 and *P*-value = 0.01. The study highlights the potential use of DNA methylation biomarkers as a non-invasive diagnostic tool for distinguishing between FCD subtypes, which could lead to more accurate treatment decisions for patients with epilepsy. The findings also underscore the importance of methylation patterns in understanding the pathophysiology of FCD.

## Teaser

Khurana *et al.* identify blood-based DNA methylation markers that mirror brain-specific changes in focal cortical dysplasia, distinguishing type IIb from IIa lesions. These findings suggest a promising, minimally invasive approach to improve diagnosis and support surgical planning in epilepsy.

## Graphical Abstract

**Graphical Abstract:**
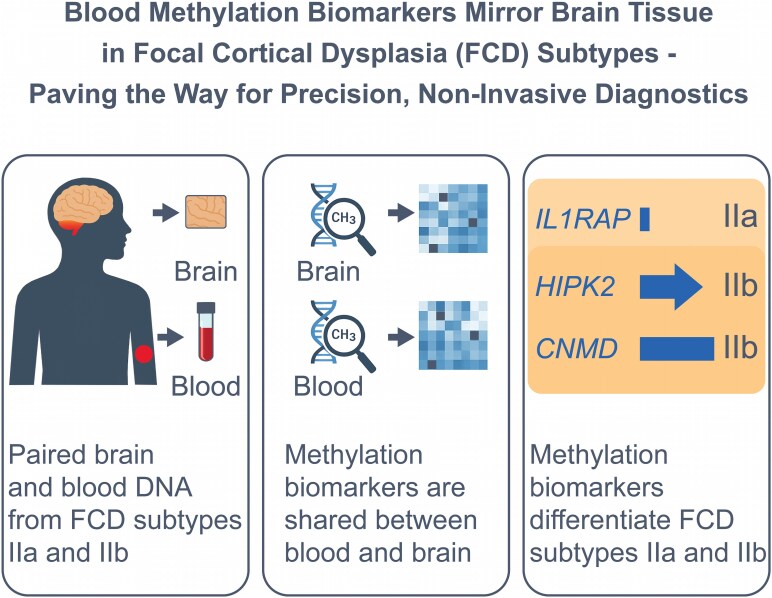

## Introduction

Focal cortical dysplasia (FCD) is a major cause of drug-resistant epilepsy in both adults and children. FCDs include a wide range of brain abnormalities that occur during the embryonic development phases. Classification of these malformations has been somewhat challenging, particularly with regard to FCDs, requiring several multidisciplinary consensuses to improve interobserver reliability in histo-pathological diagnosis.^1-5^ The mechanism(s) of their formation is not entirely understood; however, germline and somatic mutations affecting genes involved in developmental pathways have been associated with FCDs.^6-9^ FCDs vary in terms of their phenotypic presentation, which is believed to reflect the genes involved and the timing of the mutation. Some FCDs can be difficult to identify on imaging due to subtle, non-specific architectural disorganisation or cellular dysmorphism.^3,4^

Recent studies suggest that identification of the specific genetic mutation (when present) can alter clinical management and affect post-surgical outcome.^10-12^ The most recent ILAE classification of FCDs introduced a tiered approach for the management of FCDs in patients with epilepsy that integrated genetic testing with electro-imaging-clinical data in the classification scheme.^5^ Some of the most relevant genetic causes of brain FCDs include gain-of-function variants in the mechanistic target of rapamycin (MTOR) pathway (AKT3, PIK3CA, RHEB), as well as germline and somatic double-hit loss-of-function variants in its repressors (DEPDC5, TSC1, TSC2) as a substrate for FCD II and hemimegaloencephaly.^12,13^ More recently, mild malformation of cortical development with oligodendroglial hyperplasia (MOGHE) has been associated with a somatic mutation in the SLC35A2 gene seen in 40–60% of the cases.^14,15^

Modification of gene expression is coordinated by complex regulatory mechanisms that involve transcription factor binding and chromatin modification, including DNA methylation. Epigenetic modifications, such as DNA methylation, play a crucial role in altering gene expression without changing the underlying DNA sequence. The implication of epigenetic alterations through DNA methylation has been described in several neurological diseases such as Alzheimer's disease^16-18^ and brain tumours.^19-22^ Furthermore, methylation profile is a valuable tool for diagnosis, prognosis, and treatment of brain tumours.^23^

Studies in epileptic animal models^24,25^ and human brain tissues from patients with epilepsy^26,27^ have shown distinct DNA methylation patterns and epigenetic markers compared with controls suggesting that dysregulation of gene expression may contribute to the pathogenesis of epilepsy.^24,26,27^ A DNA methylation signature for chronic epilepsy has been postulated by prior research work.^28,29^ Moreover, DNA methylation analysis of brain tissue has revealed certain signatures for major FCD subtypes^30^ as well as other brain malformations such as polymicrogyria.^31^

This study examined specific gene methylation patterns within pathological samples from various subtypes of FCDs as a critical step in the quest to understand the pathophysiology of these conditions. This identification process is essential for explaining the variability in phenotypic presentations and for advancing the diagnosis and management of FCDs. It also facilitates the development of more precise therapeutic interventions tailored to the individual's genetic landscape. Given the challenges in obtaining brain tissue for FCD diagnosis, our research also aimed to assess whether leukocytes in the blood can reflect similar epigenetic changes to those observed in the brain. By examining blood DNA methylation patterns, we strive to discover a unique profile that could serve as a biomarker for pre-surgical determination of FCD type. This approach has the potential to significantly impact the clinical pathway, offering a non-invasive diagnostic tool that could lead to earlier and more accurate treatment decisions.

## Materials and methods

### Study design and participants

This retrospective study was approved by the Institutional Review Board at the Cleveland Clinic. We randomly selected 21 patients with or without drug-resistant focal epilepsy (separated into a group with FCD types I, II and III and a group with other pathologies), pathologically confirmed from our Epilepsy Biorepository, who had undergone epilepsy surgery between January 2010 and December 2023 and had matched brain and blood samples. The distribution of FCD subtypes was as follows: Ia (*n =* 1), IIa (*n =* 4), IIb (*n =* 4), IIIa (*n =* 3), and IIId (*n =* 1), with other pathologies patient group comprising the remaining (*n =* 8), as detailed in Table 1. The other pathologies included mild malformation of cortical development with heterotopic neurons^5^ (mild MCD; *n =* 2), mild malformation of cortical development with oligodendroglial hyperplasia (MOGHE; *n =* 3), polymicrogyria (PMG; *n =* 1), and no MCD (*n =* 3). We extracted the following clinical and demographic data of interest from the patient's electronic health records: age at time of surgery, gender, age at seizure onset, duration of epilepsy, surgery lobe, pathologically confirmed FCD subtype, and post-surgical seizure outcome. We also obtained the results of germline and somatic (brain tissue) genomic profiling. MRI results were also extracted from the patients’ medical records and were coded as a binary variable describing the presence or absence of an abnormality that would explain the patient's presentation. Seizure outcome was classified using Engel classification at the time of the last clinical follow-up.^32^ Post-operative outcome was considered favourable if the patient was completely seizure-free (Engel I).

**Table 1:** Clinical summary of the discovery cohort

	Focal cortical dysplasia													Other pathologies							
Classification	Ia	IIa				IIb				IIIa			IIId	mild MCD		MOGHE		PMG	non-MCD		
Paired Blood	Yes	Yes	Yes	Yes	Yes	Yes	Yes	Yes	Yes	Yes	Yes	Yes	Yes	Yes	Yes	Yes	Yes	Yes	Yes	Yes	Yes
Gender	M	F	M	F	M	F	F	M	F	F	F	F	F	M	F	M	F	F	F	M	M
age on onset (yrs)	12	4	8	0	2	13	13	5	2	17	22	25	2	5	23	0	20	6	0	49	36
Epilepsy duration (yrs)	19	14	50	1.6	19	28	25	24	39	27	44	YE	17	16	34	8	28	28	37	53	49
Laterality	R	R	R	R	L	L	L	R	L	L	L	R	R	L	R	L	R	L	R	L	L
Localisation	P	T	F	TPO	F	F	F	PO	T	T	T	T	F	F	T	F	F	F	P	T	T
Outcome (Engel score)	2	1C	1A	2A	1A	1A	1A	2	1A	4	1A	1A	1B	2	1A	N/A	1A	1A	3	1A	3
MRI (positive/negative)	+	−	−	N/A	+	+	+	+	+	−	+	+	+	−	−	+	+	+	+	−	+
*MTOR* (germline)	NT		NT	NT		NT	NT		+												NT
*NPRL3* (germline)	NT		NT	NT		NT	NT											+			NT
*SLC35A2* (somatic)	NT		NT	NT		NT	NT									+					NT

Abbreviations: F, Frontal; L, Left; NT, not tested; P, parietal; PO, parieto-occipital; R, Right; T, temporal.

Other pathologies—mMCD, MOGHE, non-MCD, PMG.

Engel outcome score: 1A (completely seizure free since surgery), 1B (Auras only since surgery), 1C (Some seizures after surgery but none for at least 2 years), 2A (initially seizure free but has rare seizures now), 2B (rare seizures since surgery), 2C (More than rare seizures initially but rare seizures for at least 2 years), 3 (worthwhile improvement), 4 (no worthwhile improvement).

Patients were tested for somatic and germline mutations (WES on known genes with germline pathogenic variants, known lesional genes with somatic variants).

### Histopathological characterization of the tissue

Surgical resected tissue samples were stored in the department of clinical pathology of the Cleveland Clinic. The neuropathological workup of surgically resected human brain tissue sections included established staining protocols, such as cresyl-violet-Luxol-fast-blue, and immunohistochemical analyses based on standardized antibody immunoreactivities published by the ILAE.^33,34^ Immunohistochemical staining was performed using the following markers: NeuN (neuronal nuclei), non-phosphorylated neurofilament protein (SMI-32), vimentin, and Map2 (microtubule-associated protein 2). The sections were reviewed by a neuropathologist (IB) for pathological classification following the guidelines of the ILAE.^5^

### DNA isolation and fragmentation

Genomic DNA (gDNA) was extracted from microdissected fresh-frozen brain tissue that included both cortical and adjacent white matter regions resected during epilepsy surgery. A 5 mL venous blood sample was collected from each participant. DNA isolation was performed using column-based purification with a phenol-chloroform-based method. Subsequently, the purified gDNA (1 µg in 50 µL of TE buffer) was fragmented into a median length of 250 bp using the Qsonica sonicator (Q800R2). The sonication was performed in pulse mode, with intervals of 30 s on and 30 s off, over a duration of 10 min at 75% amplitude, and the water temperature was maintained at 4°C. Samples were assessed for fragment size and uniformity by capillary electrophoresis on the Multi-NA (DNA-500 kit, Shimadzu).

### Methylation sequencing

Methyl-binding domain enrichment sequencing (Methyl-seq) was used to investigate DNA methylation (5-Methylcytosine) in patients with epilepsy due to various FCD subtypes and other pathologies. Methylation enrichment was performed using MethylMiner™ (Cat# ME10025, Thermo Fisher Scientific) and included NucleoMag NGS Clean-up (Cat# 744970, Macherey-Nagel). The eluted methylated-DNA was quantified, and 10 ng of this methylated DNA and matching input DNA was used to generate Illumina sequencing libraries. We used the NEBNext® Ultra™ DNA Library Prep Kit (New England Biolabs, Cat# E7370S) and indexed with NEBNext® Index Primer Set 1 (Cat# E7335S). Following library preparation and 10-plex pooling, high-throughput sequencing was carried out using the Illumina Novaseq platform (paired-end 150 bp). Input-DNA was also sequenced as a reference for methylated peak calling.

### Methyl-seq data mapping and peak calling

Raw Input-DNA and methyl-seq reads per sample were examined for quality assurance. Briefly, Fastx (version 0.0.13) quality trimmer was used to remove low-quality bases from the 3′ end of the sequence read at a base quality threshold of 20. After data quality control, the sequenced reads were aligned to the human reference genome (hg38) using BWA-MEM^35^ with default alignment parameters. Subsequently, duplicate reads were removed, and the sequence alignments for each contig were quantified, resulting in a median of 83 million reads per sample.

Following alignment, peak calling was conducted on the BAM files generated from the methylated DNA reads, which involved comparing profiles between all possible pairs of samples using the MACS peak calling software (version 2.1.1).^36^ Only peaks called with a fold-enrichment >4 and *P*-value <0.01 were retained from each sample. The selected peaks were then merged into a consensus peak set using Bedtools multiinter tool.^37^ We further filtered consensus peaks to avoid likely false positives by only including those peaks overlapping more than 2 samples and excluded peaks overlapping ENCODE blacklisted regions.^38^ For the genomic annotation of peaks, we used a custom python script to compute CpG counts within the regions and gene annotation, was obtained from Genecode (release 14)^39^ and was used to assign regions to genes based on the nearest genebody overlap. Next, ChIPseeker^40^ was utilized to assign genomic information such as region length, identifying the nearest genes and overlap with functional elements from genome assembly: GRCh38.101. Finally, the annotated peaks were quantified per sample (in both methylated DNA and input BAM files) using Bedtools multicov. The resulting count matrix was then filtered for peaks with a mean read coverage of >10 to account for potential artefact regions.

### Differential analysis

The count matrix was used as the input file for the R package edgeR,^41^ which employed a Generalized Linear Model (GLM) fitted for biological and technical variation to identify Differentially Methylated Regions (DMRs). To address inter-sample variations, methylation data was corrected for biological variability associated with age, gender, age at seizure onset, disease duration, localisation, risk factors such as family history, stroke, tumour, infection, and traumatic brain injury (TBI) and previous surgery, which were also integrated into the GLM. Any technical variances were adjusted by incorporating the enrichment ratios (calculated for each sample) of the ‘spike’ in control-methylated-DNA as part of the modelling. Additionally, to account for differences in cell heterogeneity, we employed a custom reference-based cell-type deconvolution algorithm (implemented in R) originally developed by Houseman *et al*. in 2012.^42^ This method leverages cell-type specific DMRs to estimate cell type proportions. We applied this analysis to our data and corrected the methylation data for variance associated with cell-type markers for three major types of white blood cells (B-cell; CD19, T-cell; CD3D, Monocyte; CD14).

DMRs were identified by conducting comparisons between control and case groups using edgeR. The contrasts included were: FCD I, II and III versus other pathologies, FCD subtypes versus other pathologies, FCD Ia versus other FCD subtypes, FCD IIa/IIb versus other FCD subtypes, FCD IIIa/d versus other FCD subtypes, FCD IIa versus FCD IIb, and FCD IIIa versus FCD IIId, with a significance threshold set at a *P*-value < 0.01. Next, DMRs identified by edgeR were evaluated using Receiver Operating Characteristics (ROC) analysis, conducted with the ROCR package in R.^43^ This analysis employed a binary classification model for individual DMRs to classify FCD subtypes from other pathologies. The AUC was calculated to evaluate the efficacy of DNA methylation in accurately differentiating FCD subtypes. A higher AUC indicated a better model for identifying individuals FCD subtypes. The performance of clinical risk factor modelling for ROC analysis was assessed using the Modern Applied Statistics with S (MASS) package in R^44^ [DOI; https://doi.org/10.1007/978-0-387-21706-2]. The initial model for FCD subgroup classification included age, gender, age at seizure onset, disease duration, localisation, previous surgery and combined risk factors. The stepAIC() function was employed for covariate selection, which resulted in age, gender, and combined risk factors remaining in the model. Consequently, these clinical indices were evaluated for their capability in classifying participants with FCD, in particular FCD IIb from IIa by ROC analysis.

### Methyl-qPCR biomarker validation

Next, we validated our candidate methylation biomarkers in a larger of cohort of patients with or without drug-resistant focal epilepsy, categorized by FCD IIa, FCD IIb and other pathologies. This replication cohort included paired brain and blood DNA samples (*n =* 32), as well as unpaired brain (*n =* 29) and blood (*n =* 13). Methyl-binding-domain capture quantitative PCR (Methyl-qPCR) was used to calculate percentage methylation for each sample by comparing amplification of the target sequences in unbound (unmethylated) and bound (methylated) fractions enriched after Methylminer assay. Methylated-control primer was used to amplify the methylated DNA spike-in added to each sample, assisting in normalising any technical variability between qPCR experiments (housekeeping control). SYBR Green quantitative PCR was performed with the Applied Biosystems QuantStudio™ 7 Pro Real-Time PCR system. 5 pmoles of forward and reverse primer, 4 µL of DNA template and Power SYBR™ Green PCR Master Mix (Thermofisher) were mixed to a final volume of 10 µL. Reactions were incubated at 50°C for 2 min, 95°C for 2 min, followed by 40 cycles of 95°C for 5 s and 60°C for 1 min. All RT-qPCR reactions were run in duplicates. For qPCR data analysis, threshold cycle numbers (Ct) were measured in the exponential phase for all samples and were subsequently converted into final relative values of % input enrichment. Comparisons were performed between FCD subtypes and control pathologies to identify subtype-specific methylation profiles. Data are presented as % methylation ± standard error of the mean (SEM).

### Statistical analysis

For methylation comparisons, DMRs were identified using edgeR package in R, applying a GLM approach with a significance threshold of *P* < 0.01 while accounting for biological and technical variables. ROC curves were generated using the ROCR package in R, and AUC scores were used to determine biomarker performance (AUC > 0.65, *P* < 0.01). Mann–Whitney *U* tests were applied for Methyl-qPCR validation data (Graphpad Prism 10). A *P*-value of <0.05 was considered statistically significant unless otherwise noted.

## Results

### Clinical demographics and phenotypes of FCD subtypes

Clinical, histopathological, demographic, genetic, and imaging data for the study participants is summarized in Table 1. All participants were non-Hispanic White, and most participants (62%) were female. Median age at seizure onset was 9 years (IQR = 4.5–20), and median duration of epilepsy was 27 years (IQR = 18–38). MRI imaging data was available to review in 20 of 21 patients and showed a visually identifiable lesion in 15 cases (75%). The decision to undergo surgery and the location of resection were based on the other non-invasive data (not presented here) and invasive evaluation when indicated. EEG, invasive monitoring, and other non-invasive testing were not included in this study. Surgical resection sites were as follows: frontal (*n* = 10), temporal (*n* = 7), parietal (*n* = 2), temporo-parieto-occipital (*n* = 1), and parieto-occipital (*n* = 1). The cohort included FCD subtypes Ia (*n* = 1), IIa (*n* = 4), IIb (*n* = 4), IIIa (*n* = 3), and IIId (*n* = 1), along with mMCD (*n* = 2), MOGHE (*n* = 3), PMG (*n* = 1), and no MCD (*n* = 2). Seventy percent of patients were completely seizure free (Engel I) at the time of last follow-up (median duration of follow-up is 1.3 years and *IQR* = 0.85–2.5). Genetic profiling revealed *MTOR* germline variants in two patients and *SLC35A2* somatic mutations in one MOGHE case (see Table 1 for details).

### DNA methylation profiling in epilepsy groups with or without FCD

**Figure 1:**
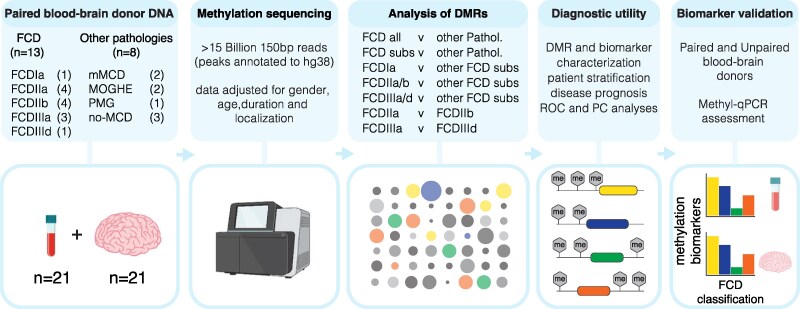
**Study framework for determining DMRs and biomarkers for subtype classification of human FCD in participants from the Cleveland Clinic.** The clinical scope and framework for determining differential methylation associated with human FCD classification in participants from the Cleveland Clinic. Genome-wide DNA methylation was analysed to identify brain and blood-based DMRs using methyl-capture coupled with deep sequencing (Methyl-seq). DMRs were identified using EdgeR for contrasts including FCD versus other pathologies, FCD subtypes versus other pathologies, FCD IIa versus IIb and FCD IIIa versus FCD IIId. Other pathologies were categorized into mMCD, MOGHE, non-MCD, and Polymicrogyria-associated epilepsy (PMG). We conducted ROC and Principal component (PC) analysis on DMRs to identify methylation (Me) based biomarkers for FCD classification for early diagnosis, patient stratification and disease prognosis. Biomarker validation was conducted via Methyl-qPCR on specific targets using paired and unpaired brain and blood samples.

This cross-sectional methylation study utilized paired DNA samples obtained from surgical brain tissues and blood samples from 21 patients diagnosed with drug-resistant epilepsy. The workflow for DNA methylation profiling in the Cleveland cohort is summarized in Fig. 1.

Paired brain and blood DNA was isolated from FCD and other pathologies and characterized for DNA methylation using MBD-capture sequencing (Methyl-seq). Our Methyl-seq approach generated an average of 250 million mapped reads per sample, totalling 15 billion reads (Fig. 1). Peak calling identified 676 551 methylated regions. These regions were mapped to ∼20 million out of 28.3 million CG sites in the human genome, providing a CG coverage of 70%. Additionally, CG site coverage was found to be similar across all groups.

**Figure 2:**
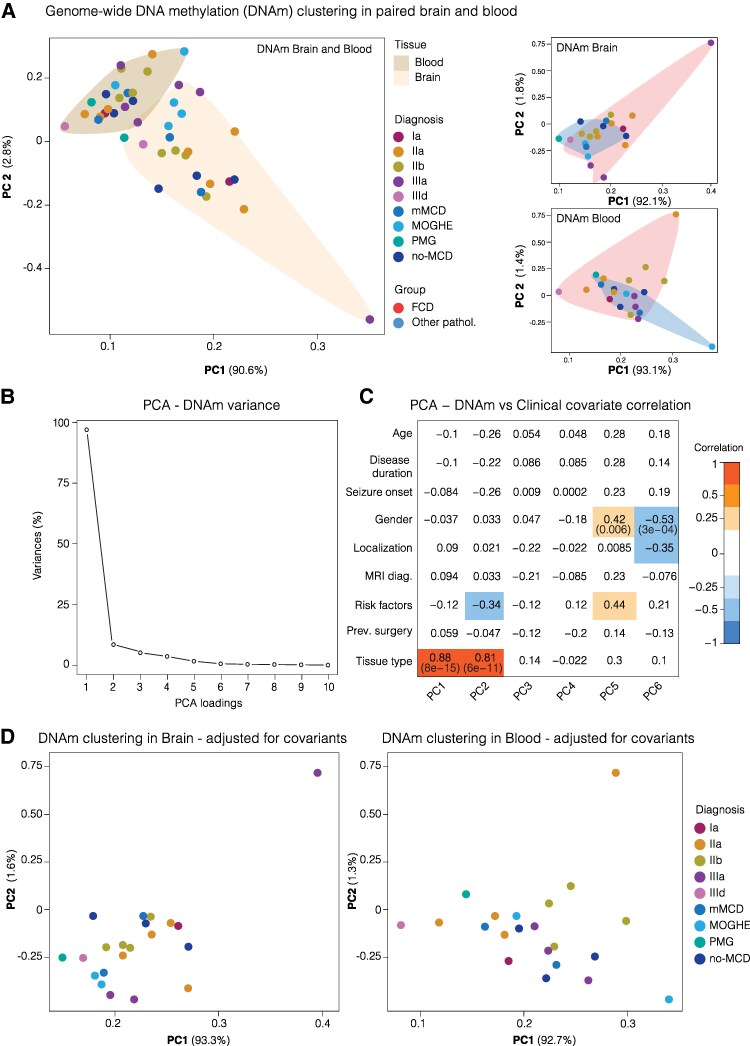
**DNA methylation clustering before and after GLM for clinical covariates.** (**A**) Principal component analysis (PCA) of unadjusted FCD Methyl-seq data obtained from paired human brain and blood DNA. Each symbol represents DNA methylation (DNAm) profile of each sample categorized by FCD subtypes (*n =* 13) and other pathologies (*n =* 8). The PCA analysis included the following: FCD Ia; patients with subtype Ia (*n =* 1), FCD IIa; patients with subtype IIa (*n =* 4), FCD IIb; patients with subtype IIb (*n =* 4), FCD IIIa; patients with subtype IIIa (*n =* 3) and FCD IIId; patients with subtype IIId (*n =* 1). Other pathologies such as mMCD, mMCD with oligodendroglial hyperplasia in epilepsy (MOGHE), non-MCD, and Polymicrogyria-associated epilepsy (PMG), were also included in the analysis. Panel 2: PCA plots show DNAm of FCD and other pathologies clustering in brain and blood DNA, respectively. (**B**) PCA variances (%) of DNAm captured by each principal component which highlights majority of the variance occurs between principal component (PC) dimensions 1–6. (**C**) Correlation between the top principal components (PC dimensions 1–6) and clinical covariates reveals the impact of clinical characteristics on genome-wide DNA methylation. The results emphasize the influence of tissue type and various clinicopathological factors, including familial history, perinatal/TBI, brain infection, brain malformation, and tumours, on DNA methylation patterns. (**D**) PCA plots of adjusted FCD Methyl-seq data clearly shows the importance of using GLM analyses to adjust for tissue type, risk factors, disease duration age variability and gender.

We observed genome-wide clustering of methylation profiles using the combined brain and blood data, as well as individual profiles for brain and blood samples before GLM adjustment (Fig. 2A). The methylation data underwent adjustments for various factors, age, gender, seizure onset, disease duration, localisation, previous surgery, cell type heterogeneity and combined risk factors, such as family history, stroke, tumour, infection, and TBI. (Figure 2B and C). PCA analyses revealed some clustering of FCD subtypes and other pathologies, for FCD IIa and IIb from the Cleveland cohort following GLM adjustment based on brain and blood methylation (Fig. 2D and E).

**Figure 3:**
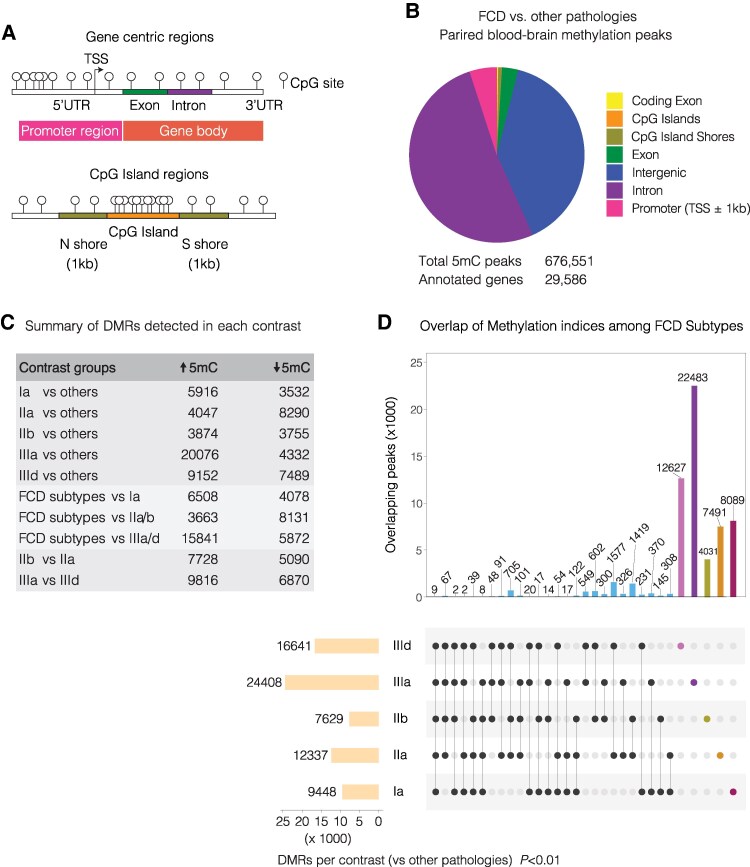
**Differential DNA methylation in human FCD and intersection among FCD subtypes.** (**A**) Schematic of gene centric regions including promoter region (±1 kb from transcription start site; TSS), genebody, CpG islands (Cytosine-phosphate-Guanine site), CpG island shores (±1 kb from CpG islands) and intergenic regions (area between annotated genes). (**B**) Distribution of methylated peaks across various genomic features including gene promoter region (±1 kb from TSS), exons, coding exons, introns, CpG islands, CpG island shores and intergenic regions. As shown, majority of the CpG differences occur at gene intronic regions. (**C**) Summary of DMRs detected in each contrast, showing number of increased and reduced methylated regions at *P*-value < 0.01 using edgeR statistical testing on paired human brain and blood DNA (*n* = 21). (**D**) Upset plot illustrates the overlap of methylation indices among different subtypes of FCD including 1A, IIa, IIb, IIIa, and IIId compared with other pathologies (*P* < 0.01). The histogram above represents the number of elements found in each subset. Each subset is ‘distinct’ and includes only those DMRs unique to the connect groups. The coloured bars indicate specific DMRs that are unique to each FCD subtype compared with other pathologies. The grid below shows the combination of contrasts that make up every subset.

DNA methylation localisation was also assessed across other genomic features, including gene promoters, gene body (exons and introns) 3′ UTR, 5′ UTR, CpG islands (CG), and CpG shores (±1 kb from CG) (Fig. 3A). Genomic mapping showed that methylation changes were primarily located in intronic regions and within gene bodies (Fig. 3B). CpG islands and shores collectively accounted for <2% of the identified differential methylation in the Cleveland cohort. We then investigated whether DNA methylation could differentiate between various subtypes of human FCD, placing specific emphasis on distinguishing FCD IIa and IIb. Pairwise comparisons of FCD subtypes and other pathologies to identify DMRs were conducted using edgeR, with a significance threshold set at *P* < 0.01, as summarized in Fig. 3C. Among these DMRs, 4047 regions exhibited increased methylation, while 8290 regions showed reduced methylation in FCD IIa compared with other pathologies. For the FCD IIb versus other pathologies group comparison, we identified 3874 regions associated with increased methylation and 3755 regions with reduced methylation. Notably, 7728 regions showed increased methylation, and 5090 regions showed reduced methylation in FCD IIb when compared with FCD IIa at *P* < 0.01.

In addition to identifying DMRs between FCD subtypes and other pathologies, our analyses included an investigation into the overlap of DMRs between different FCD subtypes. We demonstrate both shared and subtype-specific methylation signatures associated with FCD (Fig. 3D), revealing distinct DMR combinations that offer insight into the epigenetic diversity underlying FCD classifications. Overall, we identified a total of 7491 and 4031 DMRs unique to FCD IIa and IIb, respectively. This delineation of unique methylation signatures associated with each FCD subtype enhances our understanding of the methylation difference underlying the distinct pathophysiological mechanisms of FCD IIa and IIb.

### Distinguishing methylation biomarkers for FCD IIa and IIb

**Figure 4:**
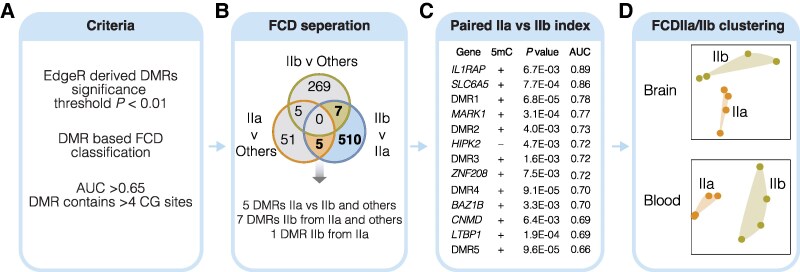
**Discriminative performance of methylation biomarkers to distinguish FCD IIa and FCD IIb.** (**A**) DMRs were identified using edgeR, with a significance threshold of *P* < 0.01, in individuals with FCD subtypes I, II and III compared with those with other pathologies from the Cleveland Clinic (*n* = 21). DMRs we assessed for classification of FCD subtypes from other pathologies, including FCD IIb versus IIa and FCD IIIa versus IIId using ROC analysis. The performance of individual DMRs in classifying case and controls was evaluated using the Area Under the ROC curve. Potential methylation biomarkers were selected based on specific criteria: AUC >0.65 per contrast analysis, DMRs with more than 4 CG sites. (**B**) Diagram of methylation biomarkers identified from contrast analyses focused on FCD IIa and IIb. The case–control comparisons included: (i) FCD IIb versus non-FCD pathologies (referred to as ‘others’), (ii) FCD IIa versus non-FCD pathologies (‘others’), and (iii) FCD IIb versus FCD IIa. The Venn diagram illustrates the intersection of DMRs for each contrast. The highlighted overlapping area represents methylation changes exclusively detected in individuals with FCD IIa and IIb, indicating their relevance to classify FCD IIa and IIb from other pathologies and further distinguish FCD IIb from IIa (blue highlight). Overall, 13 methylation biomarkers were identified for downstream assessments including 5 biomarkers which define FCD IIa from FCD IIb and other pathologies; 7 biomarkers which define FCD IIb from FCD IIa and other pathologies; and 1 biomarker was selected based on the highest AUC score in distinguishing FCD IIb from FCD IIa. (**C**) Summary of candidate methylation biomarkers (DMRs) ranked by AUC score to classify FCD IIb from FCD IIa. Data shown includes annotation to nearest genes (within 100 kb) and if not within 100 kb of gene, region is denoted as intergenic (DMR1 etc). Attributed association with gain or loss of methylation and statistical significance is also shown for each candidate biomarker. (**D**) PCA clustering of methylation biomarkers distinguishing FCD IIa and FCD IIb. The PCA plots show the clustering of samples based on the methylation signals of the 13-candidate biomarkers in paired DNA isolates from Brain tissue and Blood.

To assess the utility of DNA methylation signatures in classifying structurally similar lesions at the molecular level, specifically between FCD IIb and IIa, we conducted ROC analysis for the DMRs identified between FCD subtypes (Fig. 4A). We used a threshold of AUC >0.65, including the CG overlap criteria of >4 CG sites per region to define DMR-biomarkers. Based on these parameters, we identified a total of 61 DMRs that characterize FCD IIa and 281 DMRs that characterize FCD IIb from other pathologies samples (Supplementary Fig. 1). Furthermore, we identified 522 DMRs, which define FCD IIb from IIa with AUC >0.65 at *P* < 0.01.

To improve the discriminatory power of DMRs for classifying FCD IIb from IIa, we focused on methylation indices that overlapped across three comparisons: FCD IIa vs. other pathologies, FCD IIb vs. other pathologies, and FCD IIb vs. FCD IIa, as shown in Fig. 4B. This integrative approach identified 13 methylation biomarkers for improved differentiation of FCD IIb from IIa. Among these biomarkers, 5 candidate DMRs discriminate between FCD IIa and IIb and other pathologies, 7 candidate DMRs differentiate FCD IIb from FCD IIa and other pathologies, and 1 DMR-biomarker, with the highest AUC score of 0.89, distinguishes FCD IIb from IIa. Moreover, 8 of the 13 methylation biomarkers were located near known genes, while the remaining 5 biomarkers overlapped with intergenic regions located 100 kb away from any known genes, as detailed in Fig. 4C. The region associated with the highest AUC score (0.89) for FCD IIb classification was located within the gene body of *IL1RAP*. Notably, among the identified methylation biomarkers, only 1 exhibited loss of methylation (*HIPK2*) in FCD IIb when compared with IIa. We show the individual methylation signal of the 13 methylation biomarkers separated by FCD subtypes and ranked by AUC scores (Supplementary Fig. 2).

To underscore the diagnostic significance of the identified methylation biomarkers, we observe distinct clustering of the 13 methylation indices in FCD IIa and FCD IIb groups in paired brain and blood samples (Fig. 4D). This comprehensive analysis allows us to delineate specific methylation biomarkers associated with FCD IIb as compared to FCD IIa.

### Diagnostic value of methylation biomarkers for FCD IIa and IIb

**Figure 5:**
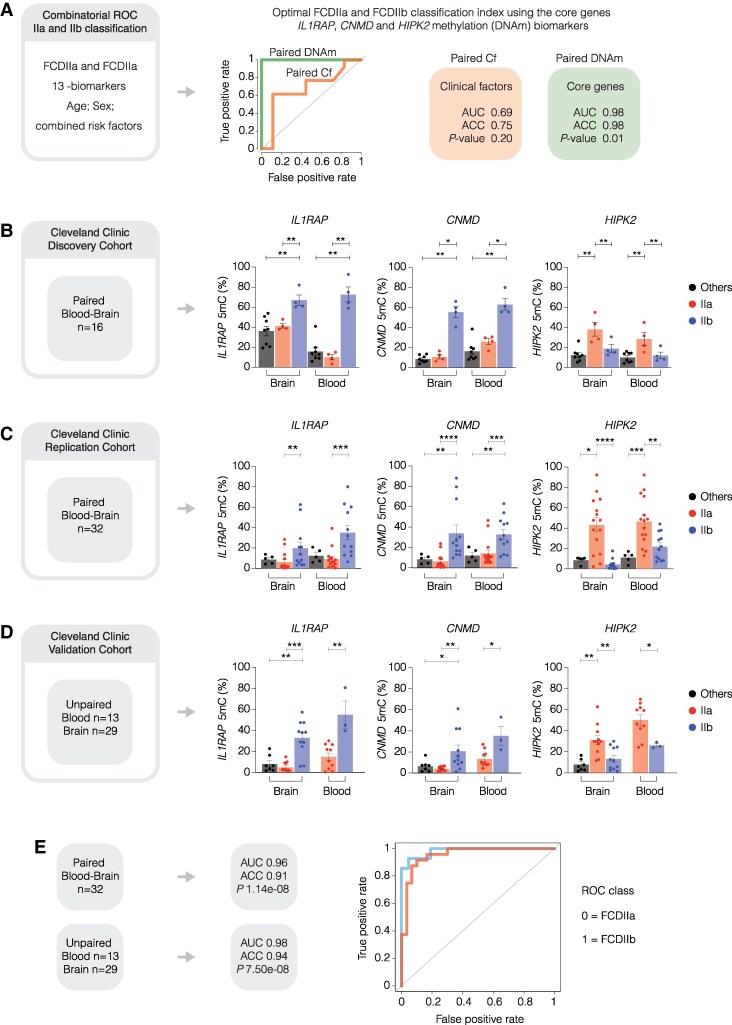
**Performance and validation of candidate biomarkers for clinical utility.** (**A**) The performance of combined DMRs and clinical risk factors in classifying individuals with FCD IIb from FCD IIa was evaluated using combinatorial ROC analysis. The combined AUC ranges for different combinations were computed, and the best combination was identified. This analysis integrates AUC scores for the 13 methylation biomarkers in paired samples from brain and blood compared with Cf to assess diagnostic performance and ACC. AUC scores were ranked for all combinations to distinguish biomarker ACC and clinical utility. Cf from the discovery Cleveland cohort (*n =* 21) were determined using the MASS package in R. The initial classification model for FCD IIa and FCD IIb groups included age, sex, age at seizure onset, disease duration, localisation, and combined risk factors, such as family history, stroke, tumour, infection, and TBI. The stepAIC() function was employed for the selection of clinical features to define FCD IIb from FCD IIa, which resulted in the best performing Cf including age, sex and combined risk factors. In the plot, the green line represents the ROC curve (AUC and accuracy) for the best-performing combination of DMRs annotated to *IL1RAP*, *HIPK2*, and *CNMD*. The orange line illustrates the AUC and accuracy for the combined clinical factors (Cf) model, which includes age, sex, and aggregated risk factors, used to distinguish FCD IIb from IIa. *P*-values were computed using bootstrap function in R. (**B**) Bar plots show the individual methylation signals for candidate DMR biomarkers (*IL1RAP*, *HIPK2*, and *CNMD*) in paired brain and blood samples from the Cleveland discovery cohort. Percentage methylation was calculated using normalized methyl-seq reads for FCD IIa (*n =* 4), FCD IIb (*n =* 4) and non-FCD pathologies (referred to as ‘others’; *n =* 8) with groups separated by tissue type. Each point represents individual sample. Statistical significance was assessed using the Mann–Whitney U test for comparisons between ‘others’ and FCD IIa, ‘others’ and FCD IIb, and between FCD IIa and FCD IIb. *P < 0.05, **P < 0.01. Error bars represent SEM. (**C**) and (**D**) Validation of FCD IIa and FCD IIb methylation biomarkers in additional samples from the Cleveland cohort. Methylation was assessed using Methyl-qPCR, a gene-specific assay to quantify DNA methylation at *IL1RAP*, *HIPK2*, and *CNMD.* The analysis included paired brain and blood DNA (*n =* 32) samples. Bar plots show methylation levels for each biomarker in in FCD IIa (*n* = 15), FCD IIb (*n* = 12), and non-FCD pathologies (‘others’; *n* = 5), separated by tissue type. For unpaired samples: FCD IIa (Brain, *n* = 11; Blood, *n* = 10), FCD IIb (Brain, *n* = 11; Blood, *n* = 3), and ‘others’ (Brain only, *n* = 7). Each point represents an individual sample. Statistical comparisons were performed as above. *P < 0.05, **P < 0.01, ***P < 0.001. Error bars represent SEM. (**E**) Combinatorial ROC analysis of *IL1RAP*, *HIPK2* and *CNMD* methylation in the Cleveland replication cohort. The ROC curve for combined DMR biomarkers in paired samples is shown in red, and the curve for unpaired samples in blue. Both assess classification performance in distinguishing FCD IIb from FCD IIa. *P*-values were calculated using bootstrap function in R.

A significant challenge is accurately defining biomarkers that interact with and distinguish between FCD IIb and IIa. These biomarkers need to exhibit high specificity and sensitivity to reliably differentiate between the two subtypes, considering the potential overlap in clinical and histopathological features. To address this, we performed combinatorial ROC analysis to prioritize methylation biomarkers for FCD IIb classification. This involved integrating the 13 methylation biomarkers with clinical factors (Cf), which generated 6884 interactive combinations to rank AUC ranges for biomarker accuracy (ACC) and clinical utility. Figure 5A shows the diagnostic performance of the methylation biomarkers when compared with the best Cf (i.e. the study covariates). The AUC score for Cf classification was 0.69 (*P* = 0.2) to define FCD IIb from IIa. The top 3 methylation biomarkers (*IL1RAP*, *HIPK2* and *CNMD*) improved the AUC score to 1 (*P* = 0.01) in classifying FCD IIb versus IIa. Individual ROC plots for these top biomarkers are provided in Supplementary Fig. 3, and the methylation signals of these biomarkers in the discovery cohort are categorized by other non-FCD pathologies (Others), FCD IIa, and FCD IIb (Fig. 5B). Specifically, increased methylation levels were observed for *IL1RAP* and *CNMD*, whereas *HIPK2* exhibited reduced methylation in FCD IIb compared with FCD IIa. These results suggest that methylation scores enhance the performance and ACC of detecting FCD IIb from other FCD subtypes, beyond what Cf alone can achieve.

### Validation of biomarkers for stratification of FCD IIb from FCD IIa

To comprehensively evaluate the specificity and sensitivity of the three methylation biomarkers (*IL1RAP*, *HIPK2*, and *CNMD*), we analysed their methylation profiles in a larger cohort (Supplementary Tables 1 and 2) recruited from the Cleveland clinic using a highly gene-specific Methyl-qPCR assay. This cohort comprised both paired (*n =* 32) and unpaired samples, including 29 brain and 13 blood samples).

We confirmed that *IL1RAP*, *HIPK2*, and *CNMD* methylation profiles in additional paired and unpaired samples from Cleveland replication cohort closely correspond to those observed in the original sequenced samples (Fig. 5C and D). Subsequently, we assessed the ROC performance of these biomarkers in the replication cohort and observed robust AUC scores of 0.96 (*P* = 1.14E−08) in paired samples and 0.98 (*P* = 7.5E−08) in unpaired samples, demonstrating their effectiveness in accurately classifying FCD IIb from IIa (Fig. 5E). Furthermore, we investigated the discriminative power of the methylation biomarkers *IL1RAP*, *HIPK2*, and *CNMD* using PCA analysis. The PCA results revealed distinct clustering of FCD IIb samples from FCD IIa samples (Supplementary Fig. 4A and B), providing additional evidence of the ability of *IL1RAP*, *HIPK2*, and *CNMD* methylation profiles to effectively differentiate FCD IIa from IIb..

## Discussion

The application of DNA methylation profiling of brain tissue has been established as an efficacious approach for the prognostication and classification of brain tumours^19,21,22,45,46^ and mild malformations of cortical development (mMCD).^47^ The diagnosis of FCD presents ongoing challenges within the field of histopathology; however, the incorporation of molecular data has the potential to enhance the differentiation of various FCD subtypes.^48^ The main objectives of this study were to identify single gene methylation candidates in the brain tissue and evaluate whether blood methylation analyses can differentiate FCD subtypes.

**Figure 6:**
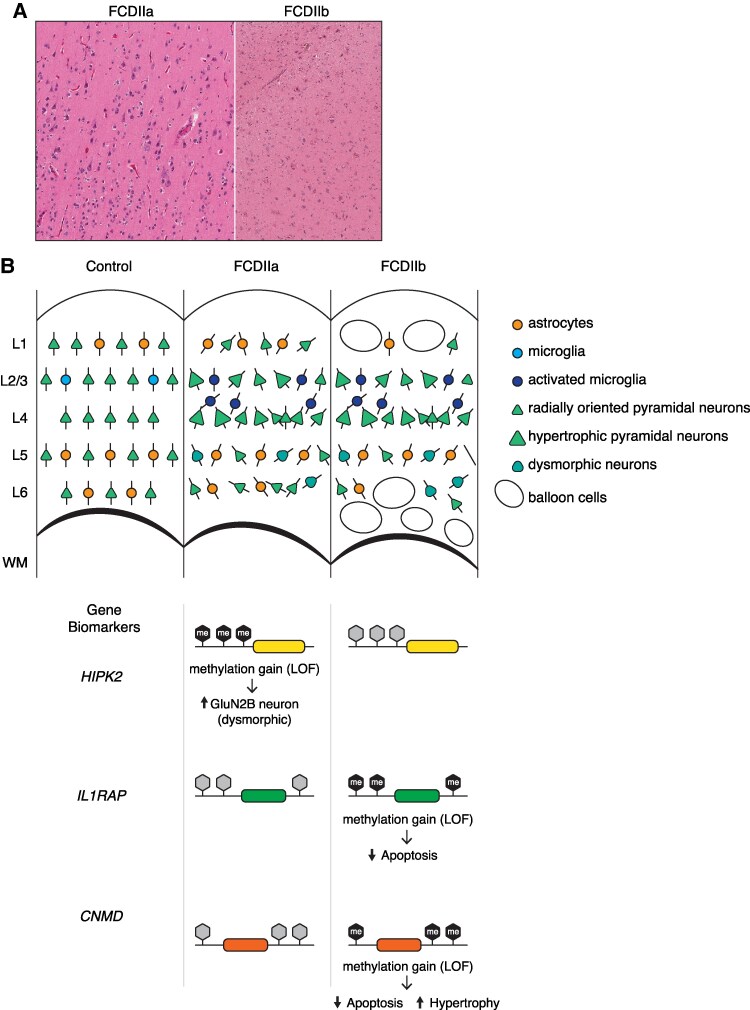
**FCD and DNA methylation biomarkers.** (**A**) Histological appearance of representative FCD types IIa and IIb tissue sections with Haematoxylin and eosin staining. (**B**) Schematic illustrating changes in DNA methylation biomarkers as a non-invasive diagnostic tool for distinguishing FCD subtypes. Proposed model of DNA methylation-dependent regulation of gene biomarkers that are associated with FCD IIa and FCD IIb. Homeodomain-interacting protein kinase 2 (*HIPK2*) gene is subject to a gain in methylation in FCD IIa to elevate GluN2B receptor expression in dysmorphic neurons. This contrasts with the loss of HIPK2 methylation is typically observed in FCD IIb. The genes coding for Interleukin 1 Receptor Accessory Protein (*IL1RAP*) and chondromodulin (*CNMD*) show methylation gains that are associated with diminished apoptosis in FCD IIb and in contrast to gene methylation in FCD IIa.

Key findings included unique and shared methylation signatures associated with each of the FCD subtypes that were present in the brain tissue as well as matched blood. We highlighted distinct DMR combinations specific to FCD IIa and IIb subtypes, helping to epigenetically define these two entities. We identified three methylation biomarkers (*IL1RAP*, *HIPK2* and *CNMD*) and validated their utility in differentiating FCD IIa from IIb in a larger replication study and an independent cohort of patients with epilepsy (Fig. 6). These biomarkers may contribute to more accurate diagnosis and targeted treatment approaches.

Although the primary focus of our study was to differentiate between FCD IIa and IIb, our methylation profiling approach also revealed distinct patterns in other FCD subtypes and non-FCD pathologies. Notably, FCD IIIa and IIId exhibited unique sets of DMRs that did not overlap with those observed in type II lesions, suggesting divergent epigenetic mechanisms underlying these subtypes. Similarly, mild malformations such as mMCD and MOGHE showed characteristic methylation signatures, including patterns associated with somatic mutations like *SLC35A2* in MOGHE cases, which were consistent with prior reports. While these groups were not the focus of biomarker validation in this study, the observed epigenetic heterogeneity underscores the potential of DNA methylation analysis in refining the molecular classification of a wide spectrum of epileptogenic lesions. Future work including larger sample sizes and subtype-specific validation will be essential to confirm the diagnostic utility of these patterns in clinical practice.

Recent studies have explored the correlation between blood and brain DNA methylation in epilepsy and other neurological disorders.^49,50^ Both studies found varying degrees of correlation between blood, saliva, buccal cells, and brain tissue methylation. Braun *et al*.^50^ reported that blood (*r* = 0.85) had high proportion of individual CpGs significantly correlated with brain, and cortical regions showing stronger correlations than the cerebellum.^47^ The presence of *IL1RAP*, *HIPK2*, and *CNMD* methylation in both brain and blood samples suggests that gene-specific blood-based methylation analysis could possibly offer a minimally invasive method for diagnosing and differentiating FCD subtypes.

Several genes implicated in epilepsy have been identified and are thought to participate in inflammation and stress, synaptic transmission and signal transduction, ion transport, cell metabolism as well as synaptic plasticity.^11-13,50^ It has been hypothesized that, not only an alteration in the sequence of nucleotides but also changes in expression of these genes through an epigenetic effect (DNA methylation) can have similar implication on the pathological and clinical phenotype, especially when these changes are sustained over the course of a long periods and particularly during stages of early development.^31,51^ DNA hypermethylation of gene bodies, introns as well as exons was shown to be associated with gene silencing in chronic rat epilepsy.^25^

Even though molecular analysis of gene expression might be needed to confirm pathogenesis of the hypermethylation seen in the involved genes, genomic mapping in our study showed that methylation changes were primarily located in intronic regions and within gene bodies. By reviewing the function of each selected gene (*IL1RAP, HIPK2* and *CNMD*), we can attempt to further understand the clinical relevance of these findings in terms of pathophysiology of FCD IIb and assess whether there is a biological relevance to the identified gene biomarkers.

Enhanced methylation of *IL1RAP* was observed in FCD IIb. *IL1RAP* is responsible for encoding the interleukin 1 receptor accessory protein, which is instrumental in the signalling cascade that activates genes responsive to interleukin 1.^52^ Interleukin1 is a pro-inflammatory cytokine but also has a crucial role in tumorigenesis and immune responses in the tumour microenvironment.^52^ Several studies demonstrated that *IL1RAP* is overexpressed in haematologic cancers^53^ In the context of gliomas, *IL1RAP* has been identified as exerting a significant inhibitory influence on these tumours by facilitating cellular apoptosis, thereby serving an anti-tumorigenic function.^54^ Concerning its potential role in neurodevelopment, experimental models have demonstrated that the absence of the two subtypes of *IL1RAP* results in reduced densities of pyramidal neurons within the cortical and hippocampal regions,^55^ implicating a possible involvement in synaptogenesis.^20^ While the specific implications of *IL1RAP* in the pathogenesis of FCD and epilepsy remain under investigation, it is hypothesized that diminished expression due to methylation may interfere with synapse formation and contribute to the anomalous proliferation of neural tissue, ultimately leading to dysmorphogenesis.

There was also increased methylation for *CNMD* that codes for a glycoprotein chondromodulin-1. Chondromodulin-I (ChM-I) 1 is a 25-kDa glycoprotein that was isolated from bovine cartilage and was shown to be a potent vascular endothelial cell growth inhibitor.^56^ It is expressed in a variety of organs, including the brain, and is known for its tumour suppressor functions in several cancers such as osteosarcomas,^18,57^ gastric adenocarcinoma^58^ and breast cancer.^59^ We can hypothesize that decreased expression of CNMD in FCD IIb through increased methylation can lead to cellular hyperplasia (i.e. Balloon cells).

*HIPK2*, the third methylation biomarker, exhibited increased methylation in FCD IIa. This pattern suggests reduced gene activity in FCD IIa. It encodes a conserved serine/threonine kinase belonging to the homeodomain-interacting protein kinase family, which interacts with homeodomain transcription factors and other regulators, including the proapoptotic activator p53. *HIPK2* is highly expressed in the central nervous system, particularly during development and early post-natal life, and plays a role in neuronal homeostasis, differentiation, and development.^60^ Its genetic ablation causes neurological defects in mice, and activation of Hipk2-p53 cell death pathway was shown to be implicated in neurodegeneration.

More recently, *HIPK2* was shown to have a role in the transcriptional control of NMDA receptor subunits GluN2A and GluN2C. NMDA receptors undergo a switch from predominantly GluN2B subunit during embryonic brain development to GluN2A subunit in early post-natal life and under-expression of *HIPK2* could potentially lead to alteration of the GluN2A/GluN2B ratio, which could prevent maturation of the NMDA receptor (persistence of GluN2B subunits) during prenatal brain development and lead to increased neurotoxicity.^61^

Our finding is consistent with previous studies that have demonstrated a sustained differential expression of the NMDA2B subunits in epileptogenic type IIa FCD tissue obtained from patients with medically intractable epilepsy.^62-64^ Furthermore, it has been established that these subunits are localized to the postsynaptic density via their association with PSD-95, a protein that anchors them to the postsynaptic site.^64^ Experimental observations have revealed an inherent increase in the excitability of FCD IIa tissue slices under magnesium-free conditions, which was selectively attenuated by the application of ifenprodil, a specific antagonist of the NMDA 2A subunit.^65^

Our results also show that the same gene-specific methylation patterns found in brain samples were observed in blood samples collected from the same patient. These findings suggest that blood-based biomarkers of methylated genes can serve as indicators of brain biology and pathology, providing a non-invasive method for disease detection and monitoring. If confirmed, these findings would lead to a pre-surgical diagnosis of the epileptic pathology (FCD IIa or IIb) in patients with MRI negative pathology, therefore optimising post-surgical outcome.

### Conclusion and future directions

Our study results provide evidence of specific single gene methylation patterns that can distinguish two common forms of FCD (FCD IIa and FCD IIb) associated with medically intractable epilepsy. These findings are found in both brain tissue and paired blood samples. These findings suggest that blood testing may be used for the detection of specific gene methylation patterns (namely *IL1RAP, HIPK2* and *CNMD)* in patients with medically intractable epilepsy suspected to result from FCD IIa or IIb lesions.

Future validation in larger and more diverse cohorts will be critical for establishing these methylation biomarkers as part of clinical decision-making in epilepsy care. Establishing a blood test to assess brain methylation changes would be a very important step in the process of identification of FCD subtypes, outcome prediction and evaluation of treatment effects (post-intervention surveillance).

## Supplementary Material

fcaf277_Supplementary_Data

## Data Availability

Raw data is not publicly available due to privacy restrictions. Data sharing is not applicable to this research project as it is independently conducted without financial support from any public entity, governmental agency, or funding body that mandates data sharing. Additionally, the resources available for anonymizing, securing, and managing shared data are limited within the scope of this study. These constraints, coupled with the necessity to safeguard participant privacy, adhere to ethical guidelines and further justify the decision to restrict data sharing in this instance. Analysis scripts used in this work (e.g. edgeR modelling, peak annotation, ROC evaluation) are provided in Supplementary Materials. These may be made available in alternative formats upon request and under appropriate transfer agreements.
