# Supplementary material for: Paired blood and brain tissue methylation biomarkers in focal cortical dysplasia
Source: Brain Commun. 2025 Aug 22;7(4):fcaf277. doi: 10.1093/braincomms/fcaf277 (PMC12371190; doi:10.1093/braincomms/fcaf277)
Supplement: fcaf277_Supplementary_Data [file fcaf277_supplementary_data.pdf]

Supplementary material for

Paired Blood and Brain tissue Methylation Biomarkers in Focal Cortical Dysplasia

This file includes:

Supplemental Tables S1 and S2 Pages 2-4

Supplemental Figures S1 to S4 Pages 6-12

R code for Differential Methylation Analysis using edgeR Pages 13-16

R code for Biomarker identification using ROC (Receiver Operating Characteristic) analysis Pages 17-20

Supplementary Table 1: Clinical data of the validation cohort with the paired blood and brain tissue.

Gender	Age onset (years)	Duration (years)	Laterality	Localization	Outcome	MRI (+/-)	Pathology
Male	0	31.9	Right	Parietal	Engel II	-	FCD IIa
Female	37	3.1	Right	Frontal	Engel I	-	No FCD
Female	17	10.9	Right	Frontal	Engel II	-	FCD IIa
Male	5	16.4	Left	Parietal	Engel I	-	FCD IIa
Male	11	11.8	Left	Frontal	Engel IV	+	FCD IIa
Female	4	36.6	Left	Frontal	Engel I	+	FCD IIb
Female	8	4	Left	Frontal	Unknown	-	FCD IIa
Female	19	46.5	Right	Frontal	Engel I	-	No FCD
Male	5.5	20.7	Right	Parietal	Engel I	-	FCD IIb
Female	0.33	18.7	Right	Frontal	Unknown	+	FCD IIa
Female	13	21.2	Left	Frontal	Engel I	-	No FCD
Male	10	12	Left	Temporal	Engel I	+	FCD IIb
Female	37	25	Left	Frontal	Engel III	-	No FCD
Male	4	24.7	Left	Parietal	Engel II	-	FCD IIb
Male	9	21.9	Left	Frontal	Engel I	-	FCD IIb
Female	11	24.8	Left	Frontal	Engel I	-	FCD IIa
Male	10	20.3	Left	Frontal	Engel I	+	FCD IIb
Male	10	9.7	Left	Temporal	Engel IV	-	FCD IIa
Male	6	11	Right	Parietal	Engel I	+	FCD IIa
Male	4	13	Left	Parietal	Engel IV	-	FCD IIa
Male	21	8	Left	Frontal	Unknown	-	FCD IIa
Male	4	17.8	Right	Frontal	Engel I	+	FCD IIb
Female	7	20	Right	Frontal	Engel I	+	FCD IIb
Female	22	3.9	Left	Frontal	Engel IV	+	No FCD
Male	7	25	Right	Temporal	Engel I	-	FCD IIa
Male	6	8	Left	Frontal	Engel I	+	FCD IIa
Male	3	2.2	Right	Frontal	Engel III-IV	+	FCD IIb
Male	0	13.7	Right	Parietal	Engel I	+	FCD IIa
Male	28	27	Left	Frontal	Engel I	+	FCD IIb
Male	5	45.2	Left	Frontal	Engel I	+	FCD IIb
Male	1.5	46.5	Left	Frontal	Engel I	+	FCD IIa
Male	2.5	13.5	Left	Frontal	Engel I	+	FCD IIb

Abbreviations: MRI +: positive MRI findings, MRI -: negative MRI findings, FCD: focal cortical dysplasia

Supplementary Table 2: Clinical data of the validation cohort with the unpaired blood and brain tissue.

Gender	Age onset (years)	Duration (years)	Laterality	Localization	Outcome	MRI (+/-)	Pathology
Female	2	22.4	Right	Multilobe	Engel II	+	FCD IIa
Female	9	10	Right	Multilobe	Engel II	+	FCD IIa
Male	2	18	Left	Frontal	Engel I	+	No FCD
Male	0.25	31.6	Right	Multilobe	Engel III	+	FCD IIb
Female	19	14.8	Right	Frontal	Engel III-IV	-	FCD IIb
Male	16	13	Left	Frontal	Engel I	+	FCD IIb
Male	5	33	Right	Frontal	Engel I	+	FCD IIb
Male	3	18	Right	Frontal	Engel IV	-	FCD IIa
Female	8	9	Right	Frontal	Engel I	+	FCD IIb
Male	8	14.2	Left	Temporal	Engel I	-	FCD IIa
Female	0.5	3.7	Left	Frontal	Engel III	+	FCD IIb
Female	10	18.8	Left	Frontal	Engel III-IV	+	FCD IIa
Female	0.92	17.3	Right	Parietal	Engel I	+	FCD IIb
Male	1	15.5	Right	Frontal	Engel III	+	FCD IIa
Female	11	2.4	Right	Temporal	Engel II	-	FCD IIb
Female	5	3.5	Right	Frontal	Engel I	+	FCD IIa
Female	0.04	2.5	Left	Frontal	Engel I	+	FCD IIa
Female	0.1	1.4	Left	Frontal	Engel I	+	FCD IIa
Female	5	3.3	Left	Frontal	Engel I	-	FCD IIa
Male	0.003	6.9	Left	Frontal	Engel IV	+	FCD IIb
Female	4	15	Right	Frontal	Engel I	+	FCD IIa
Female	12	28.5	Right	Frontal	Engel III-IV	-	FCD IIa
Male	1.75	32.3	Right	Frontal	Engel II	+	FCD IIa
Male	10	14	Right	Frontal	Unknown	-	FCD IIb
Male	0.42	0.8	Right	Frontal	Engel I	+	FCD IIa
Female	0.5	9.06	Right	Frontal	Engel I	+	FCD IIa
Female	4	16.7	Left	Frontal	Engel II	+	No FCD
Male	0.6	18.7	Left	Frontal	Engel II	-	No FCD
Female	1.75	2.9	Right	Frontal	Engel III-IV	-	FCD IIa
Male	4	20	Left	Frontal	Engel I	-	FCD IIa
Female	12	23.3	Right	Frontal	Engel I	+	FCD IIb
Male	9	28.9	Left	Frontal	Engel I	-	FCD IIb
Female	0.096	2.8	Right	Frontal	Engel II	+	FCD IIb
Female	6	43.2	Right	Frontal	Engel I	+	No FCD
Female	10	3.6	Left	Multilobe	Engel II	-	No FCD

Female	6	10.5	Left	Frontal	Engel II	+	FCD IIa
Male	17	22.6	Right	Parietal	Engel III	+	FCD IIb
Male	10	26.6	Right	Frontal	Engel I	+	No FCD
Male	4	6.9	Right	Frontal	Engel IV	-	FCD IIa
Female	10	18.8	Left	Frontal	Engel III-IV	+	No FCD
Male	38	8.5	Right	Frontal	Engel I	-	FCD IIa
Male	4	21	Right	Frontal	Engel IV	+	FCD IIa

Abbreviations: MRI +: positive MRI findings, MRI -: negative MRI findings, FCD: focal cortical dysplasia

Supplementary figure 1: Summary of differentially methylated regions of interest for distinguishing FCD subtypes from other pathologies (Others), FCD IIb from IIa and FCD IIIa from IIId after ROC analysis.

Supplementary figure 1

Identification of DMR-biomarkers per Contrast

Contrast	*Number of DMRs**
FCD Ia (v Others)	1037
FCD IIa (v Others)	61
FCD IIb (v Others)	281
FCD IIb v IIa	522
FCD IIIa (v Others)	2276
FCD IIIa v IIId	3432
FCD IIId (v Others)	2850
FCD v Others	19

**AUC > 0.65 and more than 4 CpGs per region, P <0.01*

Supplementary figure 2: Methylation signal of 13 candidate biomarkers for FCD IIb classification from FCD IIa.

(A-D) Bar plots depicting the average DNA methylation signal in individuals with FCD and those with other pathologies in the Cleveland discovery cohort. The methylation signal (methyl-seq read counts) is normalized to the input DNA and represented by the vertical axis. Each bar plot corresponds to a specific methylation biomarker in FCD subtypes and other pathologies (Others) in brain (BRN) and blood (BLD). FCD Ia (n=1), FCD IIa (n=4), FCD IIb (n=4), FCD IIIa (n=3), FCD IIId (n=1) and Others (n=8). All methylation biomarkers have a *P < 0.05 comparing FCD IIb (orange) to FCD IIa (olive Green) using the Mann-Whitney U Test. ANOVA was also conducted to compare the methylation biomarkers across multiple groups. Error bars represent the standard error of the mean (SEM) of methyl-seq reads per DMR across experiment groups. The comparison between the groups provides insights into the differential methylation patterns associated with FCD subtypes in individuals with Epilepsy.

Supplementary figure 2

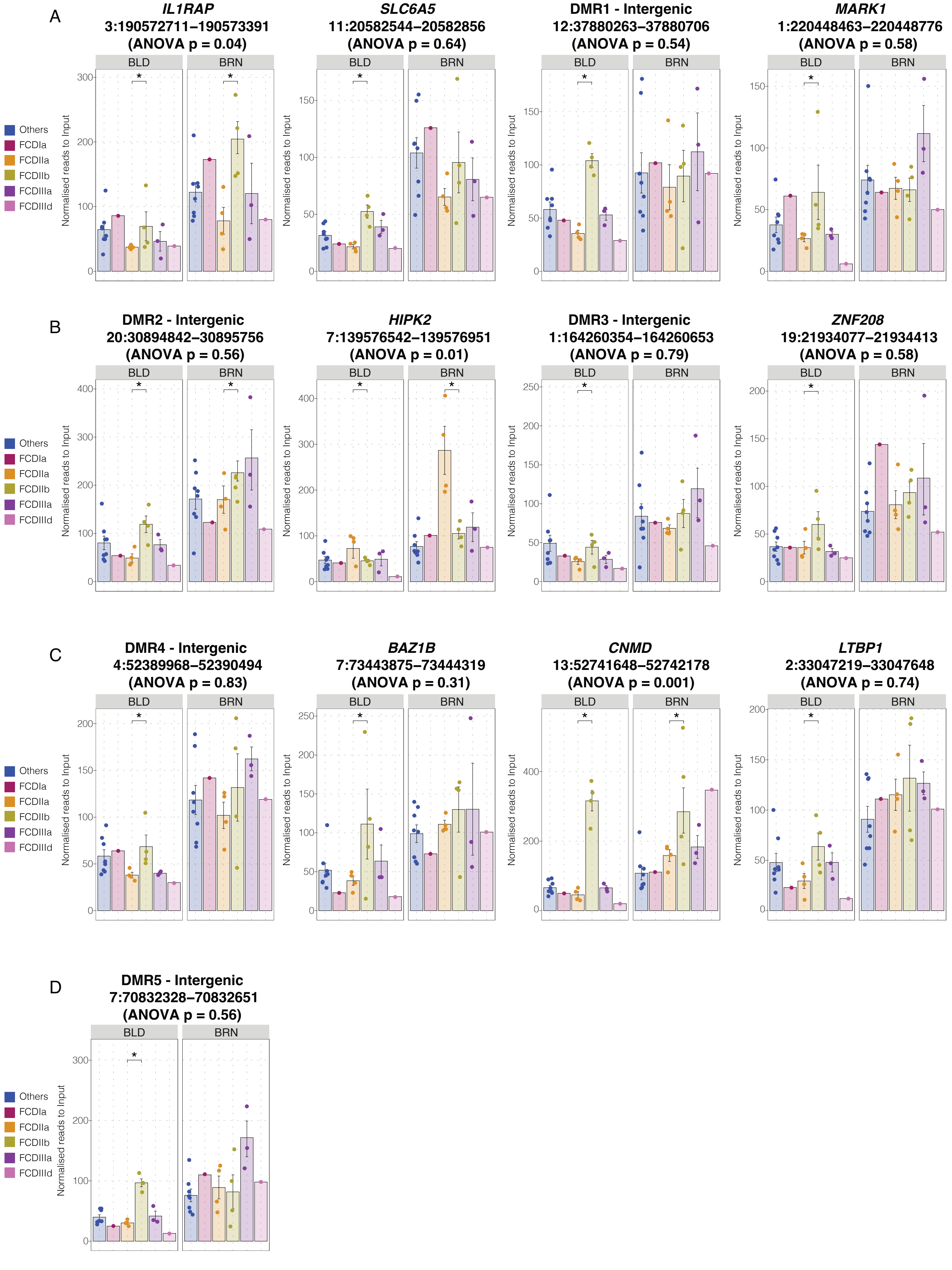

Supplementary figure 3: DMR-biomarkers for FCD IIb (vs FCD IIa) classification.

Individual ROC analysis of *IL1RAP*, *HIPK2*, and *CNMD* identified from contrast FCD IIb vs FCD IIa ($P < 0.01$). The combined area under the curve (AUC) and accuracy (ACC) of paired sample - brain (BRN, n=4) and blood (BLD, n=4) methylation is shown in black text. Blue and red text show AUC/ACC in brain and blood, respectively.

Supplementary figure 3

Performance and accuracy of individual methylation biomarkers for classification of FCD IIb from FCD IIa

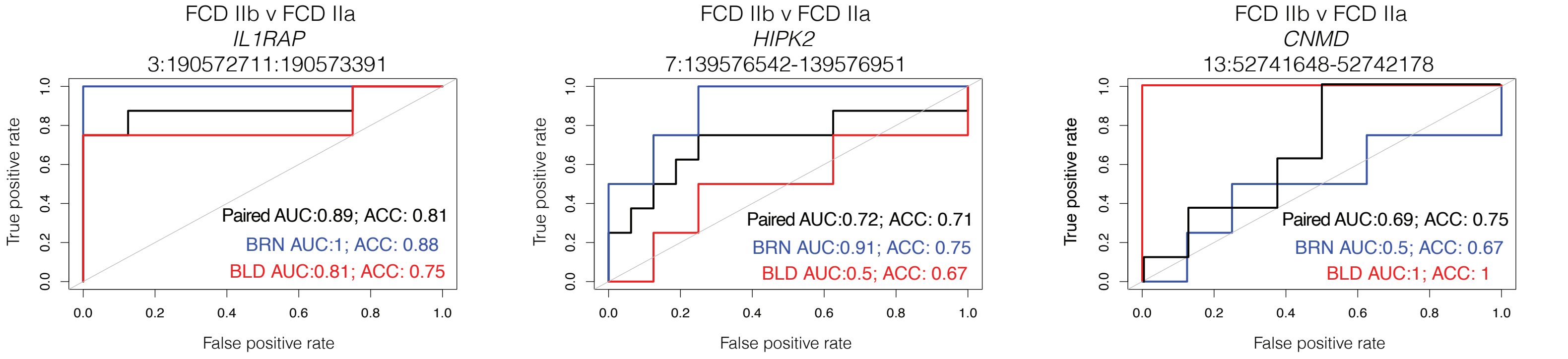

Supplementary figure 4: Clustering of FCD IIb and FCD IIa based on *IL1RAP*, *HIPK2*, and *CNMD* methylation: Brain vs Blood.

(A) PCA plots show the methylation patterns of the top biomarkers in FCD IIa (*n*= 15) and IIb (*n*= 12) groups from the Cleveland replication cohort - paired DNA isolates from brain (left panel) versus blood (right panel) for PC1 and PC2. (B) PCA plots show the methylation patterns of the top biomarkers in FCD IIa and IIb groups from the Cleveland replication cohort - unpaired DNA isolates from brain (left panel) and blood (right panel) for PC1 and PC2. Brain samples included 11 cases of FCD IIa and 11 cases of FCD IIb, while blood samples comprised 10 cases of FCD IIa and 3 cases of FCD IIb. Plots show the *IL1RAP*, *HIPK2*, and *CNMD* methylation clearly distinguishes FCD IIb from FCD IIa.

Supplementary figure 4

A

Methylation biomarkers: *IL1RAP, HIPK2, CNMD* methylation clustering

FCD IIa vs FCD IIb in Cleveland replication cohort - paired samples

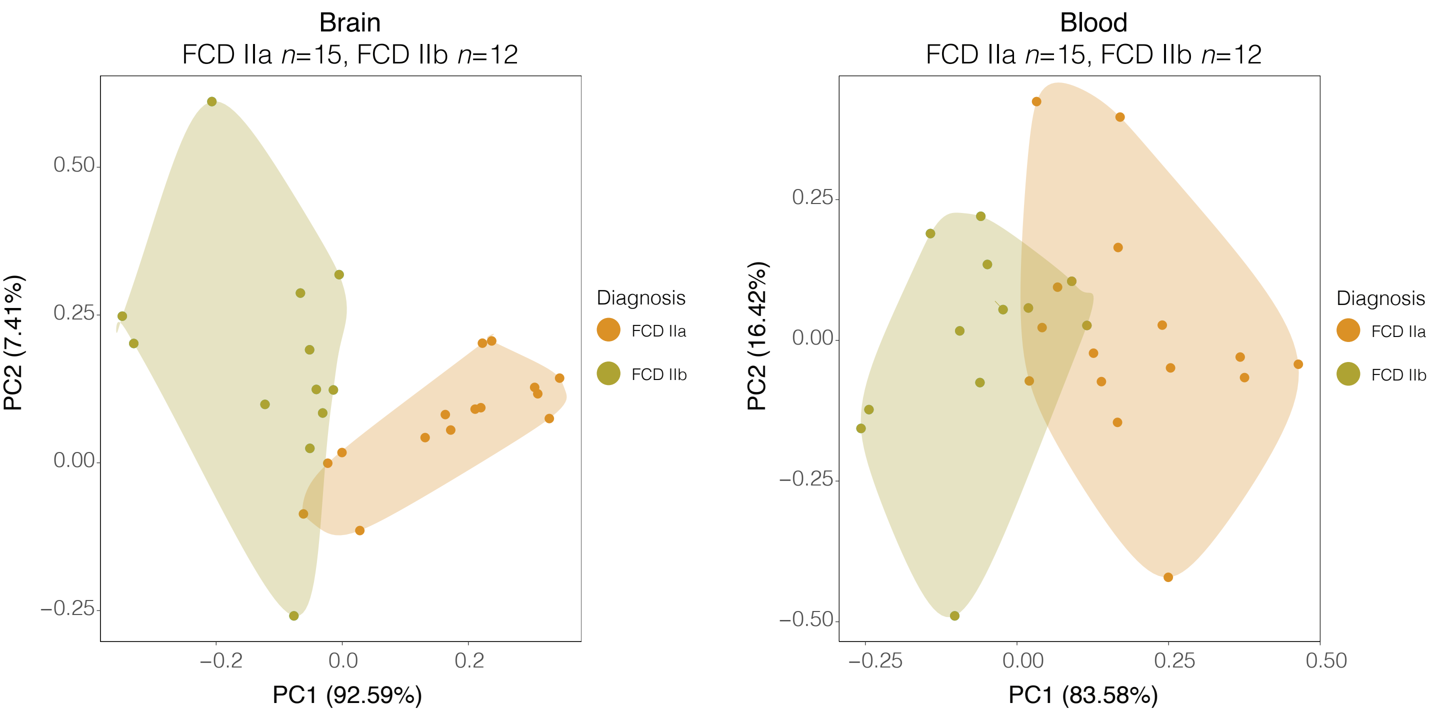

B

Methylation biomarkers: *IL1RAP, HIPK2, CNMD* methylation clustering

FCD IIa vs FCD IIb in Cleveland replication cohort - unpaired samples

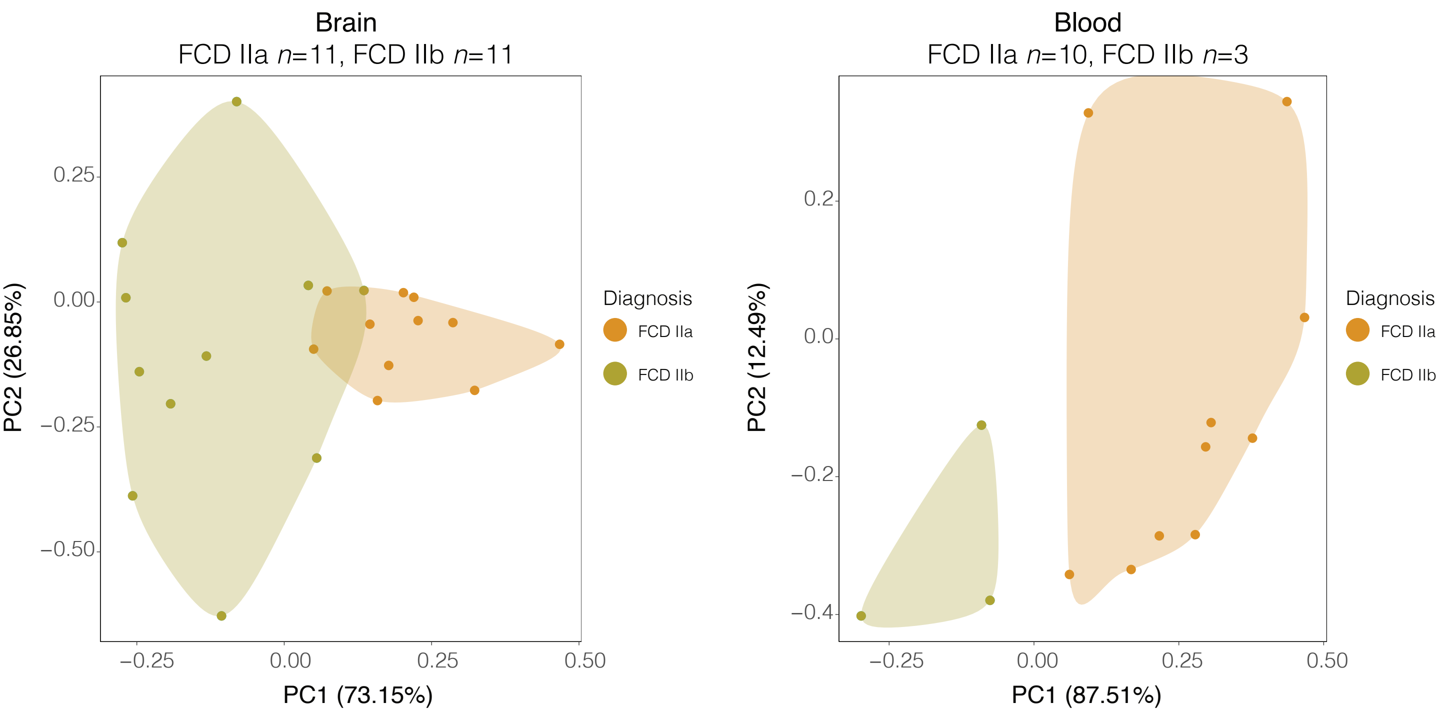

R code for Differential Methylation Analysis using edgeR

Packages required

```
library(edgeR)
library(limma)
library(sva)
library(dplyr)
library(biomaRt)
library(dplyr)
library(knitr)
library(kableExtra)
```

```
# Start code here
setwd("./project_directory")
projname <- "Cleveland_DNAm"
```

Prepare data count matrix and metadata files

```
# Read in count matrix
x <- read.table("./data/BRN_BLD_MMvIN_counts.tsv", header = TRUE, row.names = 1)

# Read and process sample metadata
CCinfo <- read.table("./data/sample_metadata.tsv.txt", header = TRUE, sep = "")
rownames(CCinfo) <- CCinfo$SAMPLE

# Subset paired samples only
CCinfo_paired_data <- subset(CCinfo, PAIR = 1)

# Define MM (methylated-DNA reads) and INPUT (reads) samples
cc <- subset (CCinfo_paired_data)
cc <- subset(CCinfo, PAIR == 1)
cc <- cc %>%
mutate(MM = gsub(".bam", "", MM), INPUT = gsub(".bam", "", INPUT))
cc_mm <- data.frame(SAMPLE = cc$MM, exp = "MM")
cc_in <- data.frame(SAMPLE = cc$INPUT, exp = "IN")
CCinfo.x <- rbind(cc_mm, cc_in)

# Basic filter: count matrix to include only paired samples and minimum average count across
samples > 10
x <- x[, CCinfo.x$SAMPLE]
x$mean <- rowSums(x) / ncol(x)
x <- x[x$mean > 10, ]
x$mean <- NULL
x <- x[rowMin(as.matrix(x)) > 0, ]
DNA.mx <- x
```

```
# Split matrix into MM and INPUT
DNA.mx.mm <- DNA.mx[, CCinfo.x$SAMPLE[CCinfo.x$exp == "MM"]]
DNA.mx.in <- DNA.mx[, CCinfo.x$SAMPLE[CCinfo.x$exp == "IN"]]
```

EdgeR modelling

#adjust for clinical covariates including, age, gender, age at seizure onset, disease duration, localization, risk factors such as family history, stroke, tumour, infection, and traumatic brain injury (TBI) and previous surgery, which were also integrated into the GLM. Any technical variances were adjusted by incorporating the enrichment ratios (calculated for each sample) of the "spike" in control-methylated-DNA as part of the modelling, denoted as "MMRO". ell heterogeneity was addressed by inputting methylation data for cell-type marker genes representing three major types of white blood cells (B-cell; CD19, T-cell; CD3D, Monocyte; CD14), denoted as "Cell.marker.MM".

```
design <-
model.matrix(~0+Diagnosis+age+gender+seizure.onset+disease.duration+risk.factors.numeric+
MMRO+Cell.marker.MM, CCinfo.mm)

# generate the GLM model of DNA methylation data using calcNormOffsetsforChIP
PRIOR = 0
D = DGEList(counts = DNA.mx.mm)
D <- calcNormOffsetsforChIP(input = DNA.mx.in, response = D)
D = estimateGLMCommonDisp(D, design)
D = estimateGLMTagwiseDisp(D, design, prior.df = PRIOR)

# fit model to groups
fit = glmFit(D, design, prior.count = PRIOR)

# Contrast Design
table(CCinfo.x$Diagnosis), specifies pathology diagnosis for each sample.
# NFCD = other pathologies

Contrast = makeContrasts(
FCD vs NFCD = ((FCD_1A + FCD_2A + FCD_2B+ FCD_3A + FCD_3D))/5 -
((mMCD)+(MOGHE) +(no_FCD)+(NoL)+(PMG)+(undet))/6,
FCD1AvFCDsubtypes = ((FCD_1A))/1 - ((FCD_2A)+(FCD_2B)+(FCD_3A)+(FCD_3D))/4,
FCD2ABvFCDsubtypes = ((FCD_2A)+(FCD_2B))/2 - ((FCD_1A) + (FCD_3A) +
(FCD_3D))/3,
FCD3ADvFCDsubtypes = ((FCD_3A)+(FCD_3D))/2 - ((FCD_1A) + (FCD_2A)+(FCD_2B))/3,
FCD1Av NFCD = ((FCD_1A))/1 -((mMCD)+(MOGHE)+(no_FCD)+(NoL)+(PMG)+(undet))/6,
FCD2Av NFCD = ((FCD_2A))/1 -
((mMCD)+(MOGHE)+(no_FCD)+(NoL)+(PMG)+(undet))/6,
FCD2BvNFCD = ((FCD_2B))/1 - ((mMCD)+(MOGHE)+(no_FCD)+(NoL)+(PMG)+(undet))/6,
FCD3AvNFCD = ((FCD_3A))/1 - ((mMCD)+(MOGHE)+(no_FCD)+(NoL)+(PMG)+(undet))/6,
FCD3DvNFCD = ((FCD_3D))/1 - ((mMCD)+(MOGHE)+(no_FCD)+(NoL)+(PMG)+(undet))/6,
FCD2BvFCD2A = ((FCD_2B) - (FCD_2A)),
FCD3AvFCD3D = ((FCD_3A) - (FCD_3D)), levels = design)
```

```
#### Run edgeR loop ####
contrast.name <- colnames(Contrast)
all_results <- list()
ranked_lists <- list()
ranked_df <- data.frame()

for (i in seq_along (contrast.name)) {
lrt <- glmLRT(fit, contrast = Contrast[, i])
disp = lrt$dispersion
fitted.vals = lrt$fitted.values
coefficients = lrt$coefficients
results <- lrt$table
results$adj.p.value <- p.adjust(results$PValue, method = "fdr")
results$adj.p.value = p.adjust(p = results$PValue, method = "fdr" )
table(row.names(results) == row.names(fitted.vals))

# Save adjusted count matrix (fitted.vals) after model
write.table(fitted.vals, file = "./data/DNA_mx_NormalisedforChIP_Adj_covars.txt", sep = "\t",
quote = FALSE, row.names = TRUE)

# Ranking scores
rank <- -log10(results$PValue) * sign(results$logFC)
rankabs <- abs(results$logFC) * rank

# Peak annotations
parts <- strsplit(rownames(results), ";")
annots <- do.call(rbind, parts)
colnames(annots) <- c("Chr", "Start", "End", "EnsID", "Gene", "CpGcount", "DMRlength",
"CpGdensity")

# Combine results
res_combined <- cbind(annots, results, rank, rankabs)
all_results[[contrast.name[i]]] <- res_combined[order(res_combined$adj.p.value), ]

# Write result table
write.table(all_results[[i]], file = paste0("./results/DGE_edgeR_", projname, "_",
contrast.name[i], ".tsv"), sep = "\t", quote = FALSE, row.names = FALSE)

# Ranked gene list
rnk <- data.frame(Gene = res_combined$Gene, rank = rank)
rnk_abs <- data.frame(Gene = res_combined$Gene, rankabs = rankabs)
rnk <- rnk[!duplicated(res_combined$EnsID) & res_combined$EnsID != ".", ]
rnk_abs <- rnk_abs[!duplicated(res_combined$EnsID) & res_combined$EnsID != ".", ]
```

```
# Save ranked lists
write.table(rnk, file = paste0("./results/DGE_edgeR_", projname, "_", contrast.name[i], ".rnk"),
sep = "\t", quote = FALSE, row.names = FALSE)
write.table(rnk_abs, file = paste0("./results/DGE_edgeR_", projname, "_", contrast.name[i],
"_ABS.rnk"), sep = "\t", quote = FALSE, row.names = FALSE)

# Collect for export
ranked_df <- rbind(ranked_df, cbind(res_combined, contrast = contrast.name[i]))
ranked_lists[[contrast.name[i]]] <- res_combined

}# End of loop
# Save combined edgeR results for differential methylation across the project
write.table(ranked_df, file = paste0("./results/DGE_edgeR_", projname, "_ALL_contrasts.tsv"),
sep = "\t", quote = FALSE, row.names = FALSE)

# Deduplicate by contrast and peak, order by rankabs
ranked_df <- ranked_df[order(abs(ranked_df$rankabs), decreasing = TRUE), ]
dedup_df <- ranked_df[!duplicated(paste(ranked_df$contrast, ranked_df$peak)), ]
write.table(dedup_df, file = paste0("./results/DGE_edgeR_", projname,
"_uniquepeaks_percontrast.tsv"), sep = "\t", quote = FALSE, row.names = FALSE)

# Filtered outputs
filtered_0.001 <- dedup_df %>% group_by(contrast) %>% filter(PValue < 0.001) %>%
ungroup()
write.table(filtered_0.001, file = paste0("./results/DGE_edgeR_", projname,
"_uniquepeaks_P0.001.tsv"), sep = "\t", quote = FALSE, row.names = FALSE)

coding_filtered <- filtered_0.001[filtered_0.001$Gene != "Intergenic", ]
write.table(coding_filtered, file = paste0("./results/DGE_edgeR_", projname,
"_top_peaks_P0.001_coding.tsv"), sep = "\t", quote = FALSE, row.names = FALSE)

# Summary tables
summary_P001 <- with(subset(dedup_df, PValue < 0.001), table(contrast, ifelse(logFC > 0,
"DMRs increased", "DMRs decreased")))
sum_P001_df <- as.data.frame.matrix(summary_P001)
sum_P001_df$Contrast <- rownames(sum_P001_df)
write.table(sum_P001_df, file = paste0("./results/DMR_summary_P0.001.tsv"), sep = "\t", quote
= FALSE, row.names = FALSE)
kable(sum_P001_df, caption = "DMRs per contrast (P < 0.001)", format = "latex", booktabs =
TRUE) %>%
kable_styling(latex_options = c("striped", "scale_down"), full_width = FALSE) %>%
save_kable(file = paste0("./results/DMR_summary_table_P0.001.pdf"))

# Save R objects
save(ranked_lists, file = paste0("./results/", projname, "_DGE_list.RData"))
save(ranked_df, file = paste0("./results/", projname, "_DGE_contrast_dataframe.RData"))
# STOP here for EdgeR
```

R code for Biomarker identification using ROC (Receiver Operating Characteristic) analysis

Packages required

```
library(ROCR)
library(knitr)
library(edgeR)
library(Biobase)
library(extrafont)
# Read-in required files
input_matrix_path <- "./data/DNA_mx_NormalisedforChIP_Adj_covars.txt"
edgeR_result_path <- "DGE_edgeR_Cleveland_DNAm_uniquepeaks_P0.001.tsv"
output_dir <- " data/biomarkers"
contrasts <- c("FCDvNFCD","FCD1AvNFCD","FCD2AvNFCD","FCD2BvNFCD",
"FCD3AvNFCD","FCD3DvNFCD","FCD2BvFCD2A","FCD3AvFCD3D")
contrast_labels <- list(
  FCDvNFCD = "FCD v other diagnosis (NFCD)",
  FCD1AvNFCD = "FCD 1A (v NFCD)",
  FCD2AvNFCD = "FCD 2A (v NFCD)",
  FCD2BvNFCD = "FCD 2B (v NFCD)",
  FCD3AvNFCD = "FCD 3A (v NFCD)",
  FCD3DvNFCD = "FCD 3D (v NFCD)",
  FCD2BvFCD2A = "FCD 2B v 2A",
  FCD3AvFCD3D = "FCD 3A v 3D"
)

# Function to read sample metadata and prepare phenotype data
pheno_data <- function(samplesheet) {
  info <- read.table(samplesheet, header = TRUE, sep = "\t")
  rownames(info) <- info$SAMPLE
  info$Diagnosis <- gsub(" ", "_", info$Diagnosis)
  info$Diagnosis <- gsub("_$", "", info$Diagnosis)
  paired <- subset(info, PAIR == 1)
  mm <- transform(paired, SAMPLE = gsub(".bam", "", MM), exp = "MM", dgsample =
gsub("MM_", "", rownames(paired)))
  list(pheno = mm[c("dgsample", "Diagnosis", "Diagnosis_class1", "FCD")], mm = mm)
}
```

```
# Main ROC analysis per contrast
run_auc_analysis <- function() {
  data_matrix <- read.delim(input_matrix_path, header = TRUE, sep = "\t", row.names = 1)
  DGE_table <- read.delim(dge_path)
  CC <- pheno_data(sample_sheet_path)
  pheno <- CC$pheno
  mm <- CC$mm
  groups <- c("NFCD","FCD 1A", "FCD 2A","FCD 2B", "FCD 3A", "FCD 3D")

output <- list()
auc_list <- list()

for (cont in contrasts) {
label <- contrast_labels[[cont]]
 group_filter <- switch(cont,
 FCDvNFCD = groups[c(2,3,4,5,6,1)],
 FCD2BvFCD2A = groups[c(4,3)],
 FCD3AvFCD3D = groups[c(6,5)],
 FCD1AvNFCD = groups[c(2,1)],
 FCD2AvNFCD = groups[c(3,1)],
 FCD2BvNFCD = groups[c(4,1)],
 FCD3AvNFCD = groups[c(5,1)],
 FCD3DvNFCD = groups[c(6,1)])

res <- read.delim(pipe(paste("grep -m 10000", cont, edgeR_result_path)), header = FALSE)
header <- read.delim(pipe(paste("head -1", edgeR_result_path)), header = TRUE)
colnames(res) <- colnames(header)

res <- subset(res, PValue < 0.001)
res$Name2 <- res$peak
expr_matrix <- t(data_matrix[res$peak, ])
colnames(expr_matrix) <- res$Name2

combined <- merge(as.data.frame(expr_matrix), pheno[, c("dgsample", "Diagnosis_class1")],
by.x = 0, by.y = "dgsample")
combined <- subset(combined, Diagnosis_class1 %in% group_filter)
combined$Diagnosis_class1 <- as.numeric(combined$Diagnosis_class1 !=
group_filter[length(group_filter)])
rownames(combined) <- combined$Row.names
combined$Row.names <- NULL

auc_scores <- sapply(combined[, -ncol(combined)], function(x) {
model <- glm(Diagnosis_class1 ~ x, family = binomial(link = "logit"), data = combined)
pred <- prediction(model$fitted.values, combined$Diagnosis_class1)
performance(pred, "auc")@y.values[[1]]
})
auc_df <- data.frame(samples = colnames(combined)[-ncol(combined)], auc =
as.numeric(auc_scores))
auc_df <- auc_df[order(-auc_df$auc), ]
```

```
output[[cont]] <- combined
auc_list[[label]] <- auc_df

}

save(output, file = file.path(output_dir,
"adj_matrix_Cleveland_biomarkers_for_ROC_pval0.001.Rdata"))

save(auc_list, file = file.path(output_dir,
"adj_matrix_Cleveland_biomarkers_for_ROC_pval0.001_AUC_LIST.Rdata"))
}

# Execute the ROC analysis
run_auc_analysis()
```

Biomarker refinement, based on AUC > 0.65 and CpG > 4

```
library(knitr)
library(dplyr)
library(kableExtra)
library(tidyr)

# Load AUC list data
load(file="adj_matrix_Cleveland_biomarkers_for_ROC_pval0.001_AUC_LIST.Rdata")

# Extract and filter biomarker info
auc_refined_list <- lapply(auc_list, function(df) {
  df <- df %>%
mutate(
DMR = samples,
peak = gsub("\\._\\.", "Intergenic_Unknown", samples),
auc = round(auc, 2)
) %>%
separate(peak, into = c("Chr", "Start", "End", "EnsID_Gene", "CpGcount", "DMRlength",
"CpGdensity_100bp"), sep = ";", convert = TRUE) %>%
separate(EnsID_Gene, into = c("EnsID", "Gene"), sep = "_", remove = FALSE) %>%
mutate(Loci = paste0(Chr, ":", Start, "-", End)) %>%
filter(auc >= 0.65, CpGcount > 4) %>%
select(DMR, peak, Loci, DMRlength, CpGcount, CpGdensity_100bp, EnsID, Gene, auc)

})
```

```
# Combine and annotate contrast name
combined_filtered_data <- do.call(rbind, auc_refined_list)
combined_filtered_data$contrast <- sub("\\.[0-9]+", "", row.names(combined_filtered_data))
row.names(combined_filtered_data) <- NULL

# Reorder columns
combined_filtered_vals <- combined_filtered_data %>%
select(DMR, peak, Loci, contrast, auc, DMRlength, CpGcount, CpGdensity_100bp, EnsID,
Gene)

# Write output
write.table(
combined_filtered_vals,
file = file.path(output_dir, "Cleveland_Biomarkers_LIST_AUC>0.65andCpG>4_p0.001.txt"),
sep = "\t", quote = FALSE, row.names = FALSE
)

# Summary table
summary_table <- combined_filtered_vals %>%
count(contrast, name = "Number of Biomarkers")

# Print table using kable
kable(summary_table,
caption = "Summary: Number of Biomarkers per Contrast (AUC > 0.65 and CpG > 4)",
format = "latex", booktabs = TRUE) %>%
kable_styling(latex_options = c("striped", "scale_down"), full_width = FALSE) %>%
save_kable(file = file.path(output_dir,
"Cleveland_summary_table_biomarkers_AUC>0.65andCpG>4_percontrast_p0.001.pdf"))
```
